# Phenotypic Shift of an Inflammatory Eosinophil Subset into a Steady-State Resident Phenotype after 2 Years of Vaccination against IL-5 in Equine Insect Bite Hypersensitivity

**DOI:** 10.3390/vetsci11100476

**Published:** 2024-10-05

**Authors:** Elio Schwarz, Fadi Jebbawi, Giulia Keller, Tanya Rhiner, Anna Fricker, Nina Waldern, Fabia Canonica, Angelika Schoster, Antonia Fettelschoss-Gabriel

**Affiliations:** 1 Evax AG, Im Binz 3, 8357 Guntershausen, Switzerland; elio@evax.ch (E.S.); fadi.jebbawi@usz.ch (F.J.); giulia.keller@usz.ch (G.K.); tanya@evax.ch (T.R.); anna@evax.ch (A.F.); nina@evax.ch (N.W.); fabia.canonica@usz.ch (F.C.); 2 Department of Dermatology, University Hospital Zurich, 8091 Schlieren, Switzerland; 3 Faculty of Medicine, University of Zurich, 8006 Zurich, Switzerland; 4 Equine Department, Vetsuisse Faculty, University of Zurich, 8006 Zurich, Switzerland; angelika.schoster@lmu.de

**Keywords:** vaccination, insect bite hypersensitivity, allergy, inflammation, equine, eosinophils

## Abstract

**Simple Summary:**

Insect bite hypersensitivity (IBH) is a common skin allergic condition in horses caused by insect bites, mainly of *Culiocides* species. Affected horses develop severe itchy lesions, up to traumatic skin injuries. Eosinophils are known to play a crucial role in the pathogenesis of IBH. Recently, we described two subsets of eosinophils: inflammatory eosinophils (iEos) dominant in blood of IBH-affected horses, and resident eosinophils (rEos) present in blood of healthy horses. iEos and rEos were distinguishable by size, granularity, and proteins present on their cell surface. Interleukin (IL)-5, the main activator and regulator of eosinophils, is our vaccine target. Vaccinated horses showed a significant reduction of total eosinophils, in particular iEos, where the very few remaining eosinophils still showed iEos phenotype. In the present study, we followed the phenotype of eosinophil subsets in the 2nd year of vaccination in IBH-affected horses. Our results showed comparably lower levels of iEos and a significant increase of rEos in 2nd compared to 1st year vaccinated and unvaccinated horses. This suggests a shift from iEos to the rEos phenotype, the dominant eosinophil type of healthy horses. The change in size, granularity and migration properties suggests a benefit of long term vaccination of IBH-affected horses.

**Abstract:**

Eosinophils play a key role in allergic diseases such as insect bite hypersensitivity (IBH). Together with Th2 cells, they shape the course of inflammation in associated type I/IVb allergies. Therefore, a virus-like particle (VLP)-based vaccine targeting equine interleukin-5 (eIL-5), eIL-5-CuMV-TT, was developed to interfere with the IL-5 dependency of eosinophils by inducing the production of anti-self-IL-5 antibodies and alleviating clinical signs in IBH-affected horses. A previous study highlighted the presence of two eosinophil subsets, steady-state resident eosinophils (rEos) and inflammatory eosinophils (iEos), circulating in the blood of healthy and IBH-affected horses, distinguishable by the expression of integrin CD49f. Furthermore, eIL-5-CuMV-TT 1st year vaccination showed a significant decrease of total eosinophils and, in particular, iEos. Nevertheless, the very few remaining eosinophils still shared an iEos phenotype, reflected by bigger size and higher granularity. The aim of this study was to follow up on the phenotype of eosinophils in the 2nd year of vaccination of IBH-affected horses with eIL-5-CuMV-TT. Using flow cytometry analysis of the blood of healthy, IBH, IBH-placebo, and IBH-vaccinated horses, the percentage and count of cells were compared between groups with a focus on pair analysis of eosinophils in 1st and 2nd year vaccinated horses. Our data showed comparably low levels of iEos and a significant increase of rEos in 2nd year compared to 1st year vaccinated horses, suggesting a phenotypic shift toward a resident-like eosinophil population, primarily associated with the phenotype of healthy horses. The reduction of size, granularity, and expression of integrin CD49f in the 2nd year suggests a benefit of long-term treatment with the eIL-5-CuMV-TT vaccine.

## 1. Introduction

Eosinophils have a recognized role in controlling parasite infections, as well as occurring as indicators of allergic reactions, and are involved in a variety of other less frequent hypereosinophilic conditions affecting various species, including humans and horses [1,2]. Their effector cell function is provided by cytotoxic granule proteins and expression of cytokines contributing to tissue homeostasis or damage, and thus they modulate adaptive and innate immunity [2,3]. Emerging from multipotent hematopoietic stem cells in the bone marrow [4], eosinophil production mainly depends on interleukin (IL)-5 [3,5,6]. Once released into the blood, eosinophils home to their corresponding tissue in various healthy organs within 8 to 18 h or they die [3,7,8,9]. In allergies, enhanced release of eosinophils from the bone marrow is caused by rising serum IL-5 levels, released by allergen-stimulated T helper (Th) type 2 cells [10]. Further, at the local site of allergic stimuli, endothelial and epithelial cells release proinflammatory cytokines and chemokines to attract eosinophils to the tissue [11], where their life span can be increased to weeks [7].

In horses, the most common allergic disease is a hypersensitivity reaction toward insect bites, predominantly caused by the genus *Culicoides* [12], called insect bite hypersensitivity (IBH) [13,14]. IBH lesions typically show notable perivascular eosinophilic infiltration together with Th2 cells in affected skin [9,13,15,16]. Moreover, the severity of IBH skin lesions has been correlated with increasing levels of eosinophils in blood [14]. Nevertheless, IBH is described as an IgE-dependent type I allergy [17,18], with eosinophils playing an important role, mostly in the late phase of type I [19,20,21] and during delayed-type hypersensitivity (DTH) type IVb allergy [14,15,22]. In the case of initial IgE dependence, it was suggested that persistent allergen exposure during the life of an allergic individual might lead to allergic chronicity of eosinophilia [23,24]. As such, conventional (c)Th2 cells might shift to IL-5-positive pathogenic effector (pe)Th2 cells [25].

When looking closer at eosinophils in the tissue of allergic reactions in mice, Mesnil et al. found that homeostatic resident eosinophils (rEos) are distinguished phenotypically and functionally from inflammatory eosinophils (iEos) [26]. Along the same lines, we recently described two distinct subsets of eosinophils in the blood of healthy and IBH-affected horses [27]. Eosinophils of healthy horses were smaller in size, contained less granules, and expressed distinctive integrin receptors compared with iEos of IBH-affected horses. Moreover, a virus-like particle (VLP)-based vaccine targeting eosinophils by inducing auto-antibodies against eIL-5 led to a significant reduction of iEos levels in the blood [27] and a significant reduction of clinical signs of IBH [14,28].

The aim of this study was to follow up on the findings on different subsets of eosinophils in IBH horses during the 2nd year of vaccination using the eIL-5-CuMV-TT vaccine. Our data confirmed a significant reduction of total eosinophil and particularly iEos counts in the blood of horses upon eIL-5-CuMV-TT vaccination in 1st year of treatment. Still, the few remaining eosinophils presented an iEos profile. Then, in the 2nd year of treatment, we observed a re-appearance of rEos, shown by small cell size, low granularity, and an integrin-expression profile on the cell surface, corresponding to the level of healthy horses.

## 2. Materials and Methods

### 2.1. Horses and Clinical Study Design

Participating horses were (i) IBH-affected horses, part of a double-blind randomized placebo-controlled study including a 2nd year half-crossover re-vaccination using the eIL-5-CuMV-TT vaccine in the 1st (IBH-VX1, n = 30)/2nd year (IBH-VX2, n = 13) or a placebo (IBH-PB, n = 30), (ii) untreated IBH-affected horses (IBH, n = 66), or (iii) unaffected healthy horses (H, n = 27) (Supplementary Table S1). The study included samples for pair analysis of the same horses in the 1st and 2nd year of vaccine treatment (n = 11).

All IBH-affected horses showed typical skin lesions on one or more parts of their body at the time of sampling, but showing otherwise normal health with frequent deworming and prophylactic vaccinations. Unaffected healthy horses were in good general condition without any history of IBH or signs of IBH at the time of examination and sampling.

The vaccine was administered subcutaneously without the presence of adjuvants [27,28]. Horses in their 1st year of vaccination received basic immunization in weeks 0 (February), 4 (March), and 12 (May), with a booster in week 24 (July/August), while horses in their 2nd year of vaccination received boosters in weeks 4 (March) and 24 (July/August). Blood collection for eosinophil subset evaluation was collected 4 weeks post injection of the vaccine or placebo at mid-season (August) during two consecutive years.

### 2.2. eIL-5-CuMV-TT Vaccine Manufacturing

eIL-5-CuMV-TT vaccine manufacturing is described in [14]. Briefly, recombinant eIL-5 protein with a C terminal linker containing a free cysteine residue and a His-Tag (eIL-5-C-His) was produced in *Escherichia coli* BL21 (DE3) cells, purified by affinity chromatography, refolded, and polished by size-exclusion chromatography. The eIL-5-C-His homodimers were then chemically coupled to a VLP based on a cucumber mosaic virus with the tetanus toxoid universal T-cell epitope tt830-843 (CuMV-TT). Free, uncoupled eIL-5 from the vaccine was removed using size-exclusion chromatography. SDS-PAGE and Western blot analysis were used to confirm the successful covalent attachment of eIL-5-C-His to CuMV-TT.

### 2.3. Blood Collection

Blood was collected from V. jugularis at the intersection of the proximal to median third of the neck using the BD Vacutainer system, a 19 G needle, and sodium heparin tubes.

### 2.4. Granulocyte Isolation

Granulocyte isolation is described in [27]. Briefly, 70 mL of blood for each horse was diluted with PBS and carefully interspersed with Ficoll^®^ Paque Plus (Merck, GE17-1440-02). After centrifugation, the sediment of red blood cells (RBC)/granulocyte pellets was collected. Lysis of RBCs was induced with sterile dH_2_O followed by the immediate addition of PBS. After centrifugation and discarding of lysed RBCs, the lysis procedure was repeated once. The sediment consisting of the isolated granulocyte fraction was resuspended in PBS.

### 2.5. Eosinophil Identification and Phenotyping by Flow Cytometry

Eosinophil identification and phenotyping is described in [27]. In summary, eosinophils were identified through specific side and forward scatter properties, followed by gating on their autofluorescence in Pacific Blue (PacBlue) and AmCyan channels. For phenotyping, cells were stained using CD49d (Anti-CD49d, PE-Cy7, Biolegend, 304314) and CD49f (Anti-CD49f, APC, Biolegend, 313616). The gating strategy with representative flow cytometry analysis is shown in Supplementary Figure S1.

### 2.6. Statistics

All statistical analyses were performed using GraphPad Prism Version 9 Software. Data were presented as mean ± standard error of the mean (SEM). The significance of the differences between several groups was determined by the Kruskal–Wallis test followed by Dunn’s Multiple Comparison post-test. Significant differences between compared pair groups were measured using the Wilcoxon test. No outliers were excluded. The appropriate statistical tests and *p* values are described in the relevant figure legends and figures, respectively. *p* values less than 0.05 were considered significant: * *p* < 0.05; ** *p* < 0.01; *** *p* < 0.001; and **** *p* < 0.0001.

## 3. Results

### 3.1. Eosinophil Cell Percentage and Counts in the Blood of Healthy, IBH, IBH-Placebo, and IBH-Vaccinated Horses

**Figure 1:**
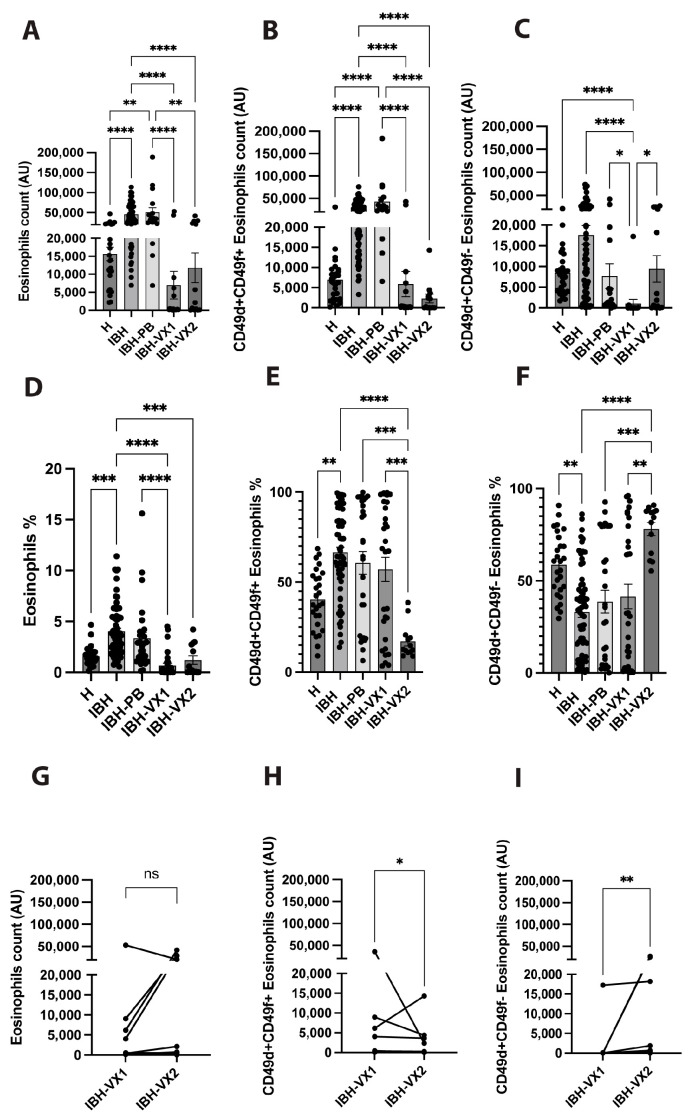
Eosinophils and surface integrin CD49d and CD49f expression. (**A**–**F**) Eosinophils of unaffected healthy (H, n = 27), IBH untreated (IBH, n = 66), IBH placebo-treated (IBH-PB, n = 30), IBH 1st- (IBH-VX1, n = 30), and 2nd-year eIL-5-CuMV-TT vaccinated (IBH-VX2, n = 13) horses. Mean cell counts of total eosinophils in absolute numbers of granulocytes (**A**) and as a percentage (**D**); double positive CD49d+CD49f+ iEos in absolute numbers of total eosinophils (**B**) and as a percentage (**E**); single positive CD49d+CD49f- rEos in absolute numbers of total eosinophils (**C**) and as a percentage (**F**). (**G**–**I**) Pair analysis of horses having received eIL-5-CuMV-TT vaccine in two consecutive years comparing 1st and 2nd year vaccination (n = 11) for cell counts of total eosinophils (**G**), double positive CD49d+CD49f+ iEos (**H**), and single positive CD49d+CD49f- rEos (**I**). *p* values less than 0.05 were considered significant: * *p* < 0.05; ** *p* < 0.01; *** *p* < 0.001; and **** *p* < 0.0001.

Blood was examined for a count of eosinophils in granulocytes with a count of the same number of events for each sample, recording eosinophils and their subsets as being either surface integrin CD49d+CD49f+ double positive (iEos) or CD49+ single positive (rEos). Eosinophil cell counts revealed a significant increase in IBH and IBH-PB horses when compared to healthy horses (Figure 1A). The eIL-5-CuMV-TT vaccination significantly reduced IBH-associated high levels of eosinophils. Hence, for IBH-VX1 and IBH-VX2 horses, eosinophil counts were found to have significantly decreased when compared to either IBH or IBH-PB horses (Figure 1A). When further subdividing the eosinophil population by surface integrin, subset counts of IBH-associated iEos were significantly increased in IBH and IBH-PB horses when compared to healthy horses (Figure 1B). Similarly to total eosinophils, IBH-VX1 and IBH-VX2 horses also showed significantly decreased iEos counts when compared to IBH and IBH-PB horses. For total eosinophil and iEos counts, there was no difference when comparing IBH-VX1 and IBH-VX2 horses. In contrast, the subset of rEos showed a significant increase in IBH-VX2 horses when compared to IBH-VX1 horses, but no significant difference to healthy horses (Figure 1C).

Additionally, taking into account the percentages of total eosinophils in granulocytes, where IBH and IBH-PB horses have a higher proportion of eosinophils than healthy horses, IBH-VX1 horses have a significantly reduced proportion of eosinophils compared with IBH and IBH-PB horses (Figure 1D). Moreover, no significant difference is found between healthy and IBH-VX1 or IBH-VX2 horses.

Further, looking at the percentages of the subsets, IBH and IBH-PB had higher proportions of iEos than healthy horses. In vaccinated horses, the small remaining iEos cell counts for IBH-VX1 horses (Figure 1B) showed a similar iEos proportion to IBH and IBH-PB horses; however, for IBH-VX2 horses, a significant drop in iEos proportion was found when comparing it to other IBH-affected horses, including IBH-VX1 horses (Figure 1E). Further, in IBH-VX2 horses the proportion of rEos rose significantly compared with IBH and IBH-PB horses as well as with IBH-VX1 horses, but showing no significant difference to healthy horses (Figure 1F).

An additional pair analysis was performed on 11 horses that were followed in the 1st and 2nd year of eIL-5-CuMV-TT vaccination. Our data showed no significant difference in total eosinophil cell counts (Figure 1G), a significant decrease in iEos cell counts (Figure 1H), and a significant increase of rEos in IBH-VX2 when compared to IBH-VX1 horses (Figure 1I).

### 3.2. Eosinophil Size and Granularity in the Blood of Healthy, IBH, IBH-Placebo, and IBH-Vaccinated Horses

**Figure 2:**
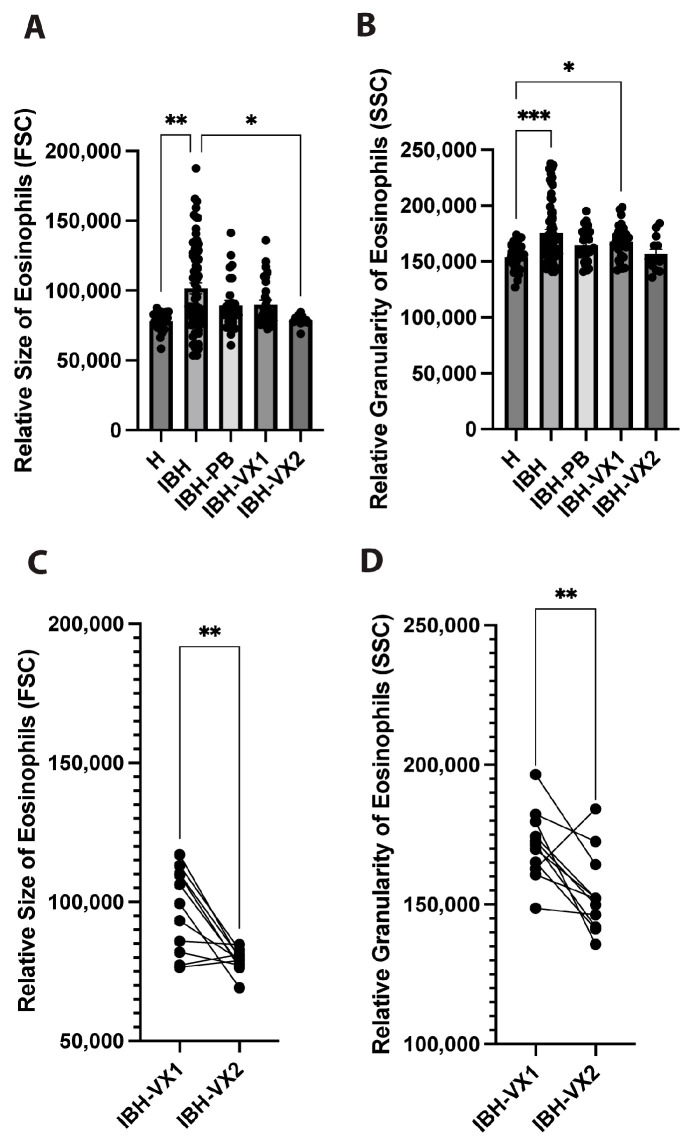
Eosinophil morphology by size and granularity. (**A**,**B**) Eosinophils of unaffected healthy (H, n = 27), IBH untreated (IBH, n = 66), IBH placebo-treated (IBH-PB, n = 30), IBH 1st year (IBH-VX1, n = 30), and 2nd year eIL-5-CuMV-TT vaccinated (IBH-VX2, n = 13) horses; flow cytometry analysis for relative size by median FSC-A (**A**) and relative granularity by median SSC-A (**B**). (**C**,**D**) Pair analysis of horses having received the eIL-5-CuMV-TT vaccine, comparing 1st and 2nd year vaccination (n = 11) using flow cytometry analysis of eosinophil relative size by median FSC-A (**C**) and relative granularity by median SSC-A (**D**). *p* values less than 0.05 were considered significant: * *p* < 0.05; ** *p* < 0.01; *** *p* < 0.001.

To morphologically characterize the eosinophils, relative size and granularity were assessed by flow cytometry forward and side scatter intensity, respectively. Eosinophils of IBH horses showed significantly increased size (Figure 2A) and granularity (Figure 2B) when compared to healthy horses. In vaccinated horses, a significant decrease in the size of eosinophils was observed in IBH-VX2 only when compared with IBH horses, but not with IBH-VX1 horses (Figure 2A).

Pair analysis for the 11 horses that were followed in the 1st and 2nd year of vaccination was performed and showed a significant decrease in size (Figure 2C) and granularity (Figure 2D) in IBH-VX2 when compared with IBH-VX1 horses.

## 4. Discussion

Recently, we showed the presence of two distinct eosinophil subpopulations in horses: rEos and iEos [27]. In healthy horses, circulating eosinophils in the blood primarily showed an rEos phenotype, with small size, low granularity, and low expression of CD49f. In contrast, eosinophils in the blood of IBH horses showed a loss of rEos and primarily showed enhanced numbers of the iEos phenotype, with relatively large size, high granularity, and high expression of CD49f. Also, our previous data showed that the 1st year eIL-5-CuMV-TT vaccination significantly decreased iEos percentage while the remaining few eosinophils predominantly retained an iEos phenotype. Here, we present a follow-up study including re-vaccinated horses, allowing a comparison of eosinophil subtypes in 1st year and 2nd year vaccinated horses. In both years, with vaccination, total eosinophil levels, and in particular iEos levels, could be significantly decreased, showing similar levels to healthy horses. In contrast to the 1st year vaccination, horses with the 2nd year vaccination showed a re-appearance of rEos, the eosinophilic phenotype of healthy horses. Hence, our data suggested a phenotypical shift from iEos to rEos in 2nd year vaccinated horses. This may suggest that long-term vaccination with eIL-5-CuMV-TT can restore rEos to normal level and phenotype in the blood of horses suffering from IBH.

Likewise, different subsets of eosinophils have been described in other species [29], e.g., in mice, and rEos and iEos were also defined [26]. In humans, different eosinophil subsets have been found, and A-Eos and B-Eos were recently defined in the gastrointestinal tract by looking at the distinction in their transcriptome, spatial localization, and surface proteome [30]. Also, earlier studies described normodense and hypodense eosinophils associated with asthma by looking at their density properties, different functionalities, and responsiveness [31,32]. Murine iEos, human A-Eos, and hypodense eosinophils show higher reactivity upon allergic stimuli [26,30,32], executing effector cell functions, which likely applies to iEos in IBH-affected horses, too [27], leaving homeostatic functions to murine rEos, human B-Eos, and normodense eosinophils and, likely, to equine rEos.

IBH horses showed significantly enhanced levels of iEos. Upon eIL-5-CuMV-TT vaccination, iEos significantly reduced to the level of healthy horses. This suggests the IL-5 dependency of iEos, which was described earlier in horses [27] and in mice [26]. The strongly decreased levels of total eosinophils in vaccinated horses, however, showed remaining marginal numbers for the iEos phenotype in 1st year vaccinated horses, which was also shown in the preceding study [27]. Interestingly, horses in the 2nd vaccination year showed a re-appearance of rEos, reflected by a reduction of size and granularity of the eosinophils and a reduction of potential migration properties shown by the downregulation of CD49f. This may suggest a switch from an iEos subtype toward an rEos subtype. While our data suggest an IL-5 dependency of iEos, the role of IL-5 on rEos in horses is not known. In mice, the role of IL-5 on rEos shows contradictory findings. While previously rEos in the lungs of mice were described as being IL-5 independent [26], a recent study suggests an IL-5 dependency of lung rEos [33]. Nevertheless, and similarly to our findings in horses, long-term treatment with an anti-IL-5 monoclonal antibody (mepolizumab) in humans restores the kinetics of eosinophils [34], allowing the possibility of subsets with different sensitivity toward an anti-IL-5 therapy.

Notably, the eIL-5-CuMV-TT vaccine previously showed an enhanced clinical effect in the 2nd vaccination year [28]. On the one hand, less variable anti-IL-5 IgG antibody titers with higher avidity may be causing such an effect [35,36]. On the other hand, in a previous study, we described a bystander reduction of basophils in the 2nd vaccination year [35], likely influencing eosinophil behavior as well. In humans, studies showed that blocking IL-5 using mepolizumab did not affect basophil counts in asthmatic patients [34], which is in contrast to therapies blocking the IL-5Ra using benralizumab [37,38]. The crosstalk between eosinophils and basophils is poorly understood, but it is suggested that basophils can have regulatory functions toward eosinophils, and probably vice versa. Basophils do express IL-5Ra [39,40] and can induce upregulation of vascular cell adhesion molecule-1 (VCAM-1) on eosinophils, which facilitates eosinophil infiltration [41,42,43,44].

Changing the levels of effector cells in allergic settings by the reduction of iEos and basophil levels, and by increasing levels of rEos in the 2nd year of eIL-5-CuMV-TT vaccination, may contribute to a sustainable and long-term benefit of the vaccine.

Of note, absolute numbers and frequencies of cells did not always match the full extend; however, this phenomenon was also observed in other studies where eosinophil frequencies and absolute numbers measured in stomach, small intestine, and colon of 8-week-old mice were controversial [45].

Understanding of eosinophils over the last decade has emphasized their multifaceted immunobiology [3,32], and several authors have noted emerging evidence for their high plasticity [29,31,46,47]. Whether or not the shift in equine eosinophil subsets from iEos to rEos after 2nd year vaccination is due to plasticity under eIL-5 vaccination or due to distinct eosinophil subsets expression remains unknown.

## 5. Conclusions

This is the first study, to our knowledge, showing the re-appearance of rEos under extended IL-5-vaccination with simultaneously reduced iEos. This may suggest a phenotypic shift from the iEos to rEos subset in eIL-5-CuMV-TT vaccinated IBH horses in the 2nd vaccination year. This further supports the vaccination’s sustainability and long-term effect in eosinophilic diseases such as IBH. Moreover, the shift in eosinophil subsets in allergic horses could represent a clinically interesting indication for determination of disease status and the possibility of interfering with iEos accumulation at an early stage, which may prevent cases of chronic IBH in horses. Further studies are necessary for a better understanding of the mechanism of action that involves basophil and eosinophil depletion, as well as eosinophil subtype shifting in the 2nd year of eIL-5-CuMV-TT vaccination of horses with IBH.

## Data Availability

The original contributions presented in the study are included in the article/Supplementary Material. Further inquiries can be directed to the corresponding author.

## Supplementary Materials

The following supporting information can be downloaded at: https://www.mdpi.com/article/10.3390/vetsci11100476/s1, Figure S1: Eosinophil identification and phenotyping; Table S1: Breed, sex, number, and age of horses included in this study.
