# Supplementary material for: Phenotypic Shift of an Inflammatory Eosinophil Subset into a Steady-State Resident Phenotype after 2 Years of Vaccination against IL-5 in Equine Insect Bite Hypersensitivity
Source: Vet Sci. 2024 Oct 5;11(10):476. doi: 10.3390/vetsci11100476 (PMC11512288; doi:10.3390/vetsci11100476)
Supplement: Supplementary file 1 [file vetsci-11-00476-s001.zip › vetsci-3176017-supplementary.pdf]

Supplementary Material

Supplementary Figures and Tables

S1.1. Supplementary Figure S1: *Eosinophil identification and phenotyping.*

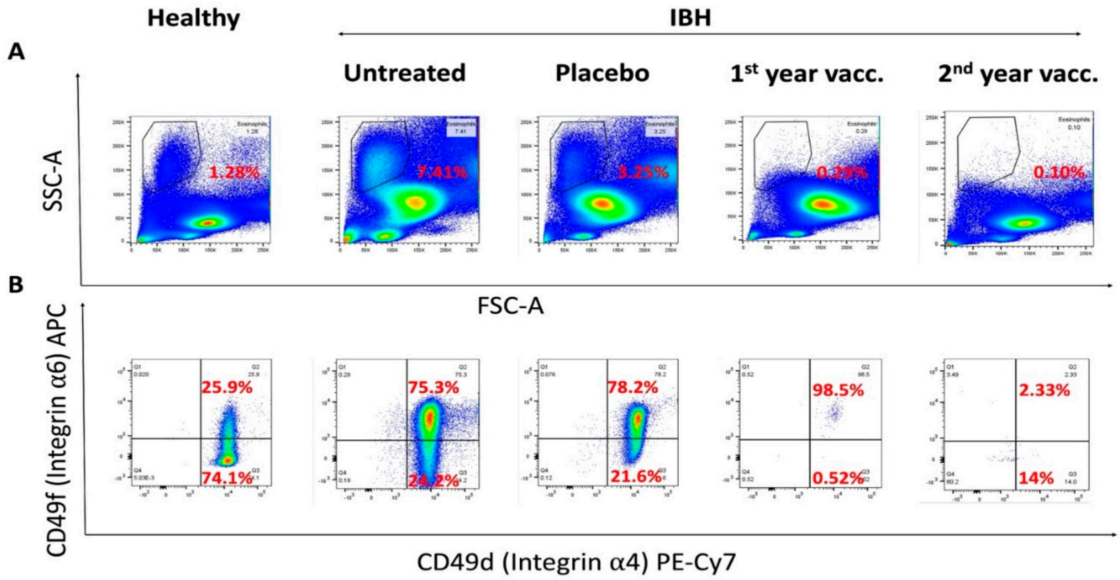

Healthy unaffected, IBH untreated, IBH placebo-treated, IBH 1st and 2nd year eIL-5-CMV$_{TT}$ vaccinated horses shown in representative flow cytometric gating on eosinophils subpopulations. **(A)** Granulocyte-enriched PBMCs shown in FSC-A/SSC-A to gate on eosinophil granulocytes. Percentage of eosinophil granulocytes marked in red. **(B)** Eosinophil granulocytes shown for CD49f and CD49d expression levels pre-gated on FSC-W/FSC-H for single cells and AmCyan/PacBlue for eosinophilic autofluorescence. Percentage of double positive CD49d+CD49f+ and single positive CD49d+CD49f- eosinophils respectively marked in red.

S1.2. Supplementary Table S1: ***breed, sex, number and age of horses included in this study***

**Groups**	**Icelandics**		**Non-Icelandics***	
	sex<br>mare / gelding / stallion	age<br>Mean (SD)	sex<br>mare / gelding / stallion	age<br>Mean (SD)
**Healthy**	3 / 10 / 2	15.47 (5.62)	6 / 6 / 0	18.42 (7.6)
**Untreated**	23 / 27 / 1	13.92 (4.79)	8 / 7 / 0	12.87 (6.01)
**Placebo**	9 / 14 / 0	16.52 (4.69)	3 / 4 / 0	12.43 (5.86)
**1st year vacc.**	8 / 15 / 0	13.96 (3.95)	3 / 4 / 0	14.14 (3.72)
**2nd year vacc.**	5 / 4 / 0	15.67 (5.32)	2 / 2 / 0	13.5 (6.45)
**Total**	48 / 70 / 3	14.74 (4.89)	22 / 23 / 0	14.53 (6.42)

* *Breed (mare/gelding)*, Arabian and partbred (1/4), Canadian horse (0/2), Freiberger (1/2), Haflinger (3/1), Hanoverian (3/0), Painthorse (1/1), PRE (3/3), Swiss warmblood (3/2), Welsh and partbred (3/0), Pony breeds (3/4), Other breeds (4/1)

Table showing study horses divided into Icelandic and Non-Icelandic horses presented by sex and age.
